# A Double-Blind Placebo-Controlled Crossover Study of the Effect of Beetroot Juice Containing Dietary Nitrate on Aortic and Brachial Blood Pressure Over 24 h

**DOI:** 10.3389/fphys.2019.00047

**Published:** 2019-02-04

**Authors:** Suraj Kukadia, Hakim-Moulay Dehbi, Therese Tillin, Emma Coady, Nish Chaturvedi, Alun D. Hughes

**Affiliations:** ^1^National Heart and Lung Institute, Faculty of Medicine, Imperial College London, London, United Kingdom; ^2^Institute of Cardiovascular Science, University College London, London, United Kingdom; ^3^CRUK Cancer Trials Centre, Faculty of Medical Sciences, University College London, London, United Kingdom; ^4^MRC Unit for Lifelong Health and Ageing at UCL, University College London, London, United Kingdom

**Keywords:** beetroot, inorganic nitrate, blood pressure, pulse wave velocity, double-blind, randomized, placebo-controlled crossover study

## Abstract

Dietary inorganic nitrate in beetroot can act as a source of nitric oxide and has been reported to lower brachial blood pressure (BP). This study examined the effect of inorganic nitrate in beetroot juice on aortic (central) BP acutely and over the subsequent 24-h period. A double blind, randomized, placebo-controlled crossover trial was performed in fifteen healthy, normotensive men and women (age 22–40 years). Participants were randomized to receive beetroot juice containing nitrate (6.5–7.3 mmol) or placebo beetroot juice from which nitrate had been removed (<0.06 mmol nitrate). Effects on aortic systolic BP were measured at 30 min (primary endpoint), 60 min and over a subsequent 24 h period using an ambulatory BP monitor. Carotid-femoral pulse wave velocity (cfPWV) was also measured at 30 min. Following a washout period, the procedure was repeated within 7 days with crossover to the opposite arm of the trial. Compared with placebo, ingestion of beetroot juice containing nitrate lowered aortic systolic BP at 30 min by 5.2 (1.9–8.5) mmHg [mean (95% confidence interval); *p* < 0.01]. A smaller effect on aortic systolic BP was observed at 60 min. There were minimal effects on brachial BP or cfPWV. Effects on aortic systolic BP were not sustained over the subsequent 24 h and there were no effects on other hemodynamic parameters during ambulatory monitoring. A single dose of beetroot juice containing nitrate lowers aortic BP more effectively than brachial BP in the short term, but the effects are comparatively short-lived and do not persist over the course of the same day.

## Introduction

High blood pressure (BP) affects up to a billion people worldwide and is an important risk factor for cardiovascular disease even at levels below those traditionally classified as hypertensive (Forouzanfar et al., 2017). In older people high BP is largely attributable to elevated systolic BP secondary to increased arterial stiffness (Franklin et al., 1997).

Dietary measures offer potential as large-scale, cost-effective, and low-risk interventions that may offer a population-based strategy to complement drug-based therapy targeted at high risk individuals. Beetroot is a rich source of dietary inorganic nitrate (Lidder and Webb, 2013). Inorganic nitrate is absorbed in the proximal small intestine and, *via* the enterosalivary circulation, can act as a source of nitrite anions and nitric oxide (Lidder and Webb, 2013). Nitric oxide, which is endogenously produced by endothelial cells, is a vasodilator with a range of vasoprotective functions (Moncada and Higgs, 1993). Tissue and blood nitrite, derived either from oxidation of nitric oxide or dietary sources of nitrite/nitrate, serves as a reservoir from which nitric oxide can be produced through acidification or *via* proteins, including hemoglobin, that possess nitrite-reductase activity (Shiva, 2013). This mechanism contributes to skeletal muscle vasodilation in response to hypoxia (Dinenno, 2016) and nitrite supplementation reduces the BP response to the metaboreflex in skeletal muscle in older adults (Schneider et al., 2018). Inorganic nitrite supplementation has also been reported to improve endothelial dysfunction and decrease arterial stiffness in aged mice (Sindler et al., 2011) and older humans with moderately elevated cardiovascular risk (Rammos et al., 2014).

Previous studies have examined short-term effects of beetroot juice on brachial BP (Larsen et al., 2006; Webb et al., 2008; Kapil et al., 2010, 2015; Gilchrist et al., 2011; Hobbs et al., 2012), but little is known about its effect on aortic (central) BP. Aortic BP has been shown to better predict incident cardiovascular disease than brachial BP (Vlachopoulos et al., 2010) and is more closely associated with vascular and cardiac target organ damage (Kollias et al., 2016). Organic nitrates, while differing in some respects from inorganic nitrite/nitrate, also act via production of nitric oxide (Omar et al., 2012) and reduce aortic more than brachial BP (Jiang et al., 2002). Whether dietary inorganic nitrate has similarly more marked effects on aortic pressure has not been studies. It is also established that ambulatory BP measured over 24 h is a better predictor of cardiovascular risk and total mortality than clinic measurements of BP (Banegas et al., 2018), and recently it has become possible to measure aortic BP over 24 h (Weiss et al., 2012).

We therefore hypothesized that dietary nitrate in beetroot juice would reduce aortic BP acutely after administration. We also aimed to examine its effect on aortic blood pressure over a 24-h period, arterial stiffness and other measures of vascular function, and to compare its effect on aortic and brachial systolic BP.

## Materials and Methods

### Participants

Fifteen healthy volunteers (eleven female and four male) consisting of medical students and members of staff of Imperial College London were recruited. Healthy participants aged over 18 years with no existing co-morbidities, including hypertension, or on any medication (other than oral contraceptive pill), were eligible for the study. Studies were performed at the International Centre for Circulatory Health, Imperial College London, United Kingdom. The study protocol was approved by the London-Fulham Research Ethics Committee, Charing Cross Hospital (Ref: 13/LO/0063). All participants gave written informed consent in accordance with the principles of the Declaration of Helsinki.

### Study Design and Interventions

**FIGURE 1:**
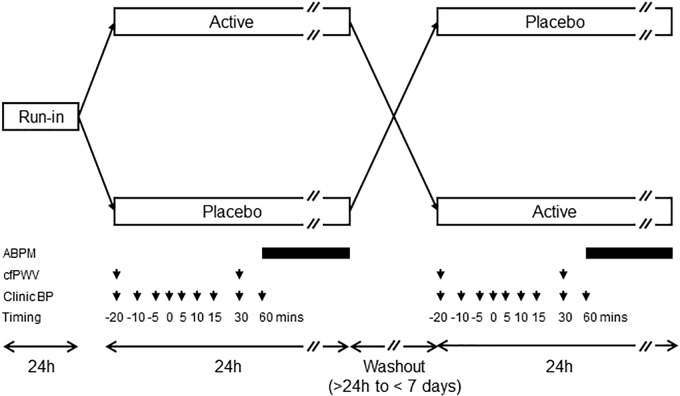
Diagram showing crossover design. Timings of measurements of clinic blood pressure (BP), carotid-femoral pulse wave velocity (cfPWV), and ambulatory blood pressure monitoring (ABPM) with respect to administration of placebo or active juice (time = 0) are indicated by the arrows and bars, respectively. ABPM was performed every 30 min during the day and hourly during the night.

This study was designed as a double-blind, randomized, placebo-controlled crossover trial (Figure 1), all observers were blinded to allocation status and participants were randomized using a computer program. All individuals were required to abstain from drinking caffeine-containing beverages alcohol and smoking for 12 h prior to study days and during the study. During the 24-h period following ingestion of the study drinks, no other dietary restrictions were imposed except that participants were asked not to consume any food or drink containing beetroot. A “washout” period of at least 24 h and less than 7 days was chosen on the basis of the duration of action of beetroot seen in other studies (Cosby et al., 2003; Coles and Clifton, 2012). Ingestion of study drinks was performed at the same time of day.

The active intervention containing dietary nitrate was administered as a 70 ml concentrated beetroot juice drink (Beet It Sport Stamina, Beet It Beetroot Products Company) which contained 6.5–7.3 mmol nitrate. “Nitrate-free” beetroot juice (containing <0.06 mmol nitrate) from the same company which was identical in appearance acted as placebo.

### Study Measurements

A questionnaire was administered to collect information on lifestyle, and personal and family medical history. Height was measured using a stadiometer and weight was measured using a Soehnle electronic scale.

Sitting clinic BP (aortic and brachial) and heart rate were measured using a Pulsecor device (Pulsecor, Auckland, New Zealand) (Park et al., 2014). Measurements of BP were made at 20, 10, and 5 min and immediately prior to ingestion of the active of placebo drink. Subsequent measurements of BP were made at 5, 10, 15, 30, and 60 min after ingestion. After the final Pulsecor measurement at 60 min, a 24 h BP monitoring device (Mobil-O-Graph^®^), which measures both aortic and brachial BP (Weiss et al., 2012), was fitted to the participant’s non-dominant arm. The Mobil-O-Graph^®^ recorded BP at 30-min intervals throughout the day, and at hourly intervals during the night.

Carotid-femoral pulse wave velocity (cfPWV) was measured using a Vicorder device (Skidmore Medical Limited, Bristol, United Kingdom) applied to the neck and right thigh of all subjects according to current guidelines (Van Bortel et al., 2012). cfPWV was measured approximately 20 min before and 30 min after ingestion of active or placebo drink. An indirect estimate of aortic pulse wave velocity (iPWV) during 24 h BP measurement was also obtained from the Mobil-O-Graph^®^ as previously described (Luzardo et al., 2012).

### Sample Size Determination and Statistical Analysis

The sample size was chosen to detect a 4 mmHg treatment difference in the primary endpoint, aortic systolic BP (alpha = 0.05; 80% power) assuming a standard deviation (SD) of 5 mmHg.

Statistical analysis and randomization was performed using R 3.0.2. Sample characteristics are presented as mean ± SD for normally distributed data or median (25th, 75th centiles) for skewed data, or n (%) for categorical data. Results are presented as the treatment effect (95% confidence interval). Data were analyzed by means of linear mixed models with random intercepts and common slopes to study the treatment effect, adjusting for baseline measures immediately prior to ingestion. The assessment of period effects and interactions between period and treatment was done within the models by including a covariate for the period in which the treatment was given, and a covariate representing the interaction between the period and the treatment. When no evidence of period effects or interactions between period and treatment was found, these covariates were excluded using an analysis of deviance, which led to a final, more parsimonious, model. Model assumptions were verified by analyzing residuals plots, and robust linear mixed models were used when assumptions were violated. All statistical tests were two-sided and statistical significance for the primary endpoint was set at *p* < 0.05.

## Results

Participant characteristics are shown in Table 1; volunteers were predominantly young females, only two individuals smoked, body mass index was in the healthy range and alcohol intake was moderate.

**Table 1:** Participant characteristics at recruitment, pre-intervention.

Characteristics	Value
Number of participants	15
Male	4 (26.7%)
Age, years	29.2 ± 8.3
Height, cm	166.0 ± 9.6
Weight, kg	67.2 ± 15.9
Body mass index, kg/m^2^	23.4 (21.3, 24.6)
Brachial systolic blood pressure, mmHg	108.1 ± 7.2
Aortic systolic blood pressure, mmHg	100.4 ± 7.4
Diastolic blood pressure, mmHg	67.8 ± 5.37
Heart rate, beats/min	64.7 (58.3, 71.3)
cfPWV, m/s	5.71 ± 0.79
iPWV, m/s	5.67 ± 0.96
Alcohol, units/week	6.1 ± 5.7
Current smoking, n (%)	2 (11.1%)


*Values are mean ± SD or median (inter-quartile range) for skewed data. Categorical data are shown as n (%).*

### Clinic Measurements

Ingestion of beetroot juice containing nitrate resulted in a lower aortic systolic BP at 30 min (primary endpoint) compared with placebo [difference (Δ) = -5.2 (-8.2, -2.2) mmHg; *p* < 0.01; Table 2]. Aortic systolic BP remained lower at 60 min after beetroot juice ingestion, although the difference was smaller [-3.2 (-6.6, 0.2) mmHg; Table 2].

**Table 2:** Treatment effects and 95% confidence intervals of clinic measurements 30 and 60 min post-ingestion of beetroot juice compared to placebo.

Variable	Treatment effect (95% confidence interval)	
	
	30 min	60 min
Aortic SBP, mmHg	-5.2 (-8.2, -2.2)^∗^	-3.2 (-6.6, 0.2)
Brachial SBP, mmHg	-0.8 (-5.3, 3.6)	-0.7 (-4.3, 2.9)
DBP, mmHg	-2.0 (-4.4, 0.4)	-0.5 (-3.0, 2.1)
cfPWV, m/s	-0.1 (-0.3, 0.2)	–
Heart rate, bpm	-2.2 (-5.9, 1.3)	-2.0 (-6.2, 2.2)


*SBP, systolic blood pressure; DBP, diastolic blood pressure; cfPWV, carotid-femoral pulse wave velocity. ^∗^Primary outcome (p < 0.01).*

In contrast brachial systolic BP did not differ noticeably between active and placebo groups at 30 or 60 min post-ingestion, BP in the active group was slightly lower [-0.8 (-5.3, 3.6)/-2.0 (-4.4, 0.4) mmHg; Table 2] but the confidence intervals of both brachial systolic and diastolic BP included zero.

There was no evidence of either a period effect or a treatment x period interaction for either aortic or brachial systolic pressure. Effects on heart rate and cfPWV are shown in Table 2. None of these measurements differed between active and placebo drink at 30 or 60 min post-ingestion.

### Ambulatory Blood Pressure Measurements

Average 24-h aortic systolic BP was -0.9 (-2.5, 0.7) mmHg (lower) after beetroot juice ingestion than after placebo but the 95% confidence interval included zero. Differences between treatments were small and confidence intervals included zero for all other ambulatory BP variables, including iPWV (Table 3). Supplementary Figures S1–S6 show the ambulatory measurements over the 24-h period, for active treatment and placebo.

**Table 3:** Treatment effects and 95% confidence intervals of 24 h ambulatory measurements after beetroot juice ingestion compared to placebo.

Variable	Treatment effect (95% confidence interval)
Aortic SBP, mmHg	-0.9 (-2.5, 0.7)
DBP, mmHg	-0.3 (-1.8, 1.3)
Heart rate, bpm	-1.90 (-3.7, 0.1)
iPWV, m/s	-0.04 (-0.10, 0.01)
Reflection magnitude, %	1.4 (-0.0, 2.7)
Augmentation index, %	-0.7 (-2.4, 0.9)


*SBP, systolic blood pressure; DBP, diastolic blood pressure; iPWV, indirect aortic pulse wave velocity.*

### Adverse Events

There were no major adverse events. All participants were asked if they experienced any headaches, flushing or light-headedness; one participant complained of headache after consuming beetroot juice containing nitrate (active). This headache lasted for approximately 10 min. No other symptoms were reported by any of the participants.

## Discussion

This randomized, double blind, placebo-controlled crossover study has demonstrated that beetroot juice containing ∼7 mmol inorganic nitrate lowers aortic systolic BP by ∼5 mmHg compared with placebo, with the peak effect occurring approximately 30 min after ingestion. The effect of beetroot juice containing inorganic nitrate was more marked on aortic than brachial systolic BP and there was little or no effect of ingestion of inorganic nitrate on subsequent 24-h aortic BP.

Previous studies have examined the effect of dietary nitrate on brachial BP. Kapil et al. (2010) found that, compared with water, beetroot juice containing 5.5 mmol nitrate caused a peak reduction in brachial systolic BP of ∼5 mmHg at 3 h with no change in diastolic BP. It is possible that differences in drink composition or participant characteristics contributed to the greater effect on brachial BP seen in their study. For example, participants in their study typically had brachial systolic BP of ∼120 mmHg compared with 110 mmHg in our study and the effect of hypotensive agents tends to be greater with higher baseline BP (Messerli et al., 2015). Also, ingestion of water has been reported to increase brachial BP (Jordan et al., 2000), probably through a mechanism involving the sympathetic nervous system and elicited by hypo-osmolarity (May and Jordan, 2011); while this effect appears small in young individuals (Jordan et al., 2000) it may mean that water is an unsuitable control for beetroot juice. In another study, Coles and Clifton compared a beetroot and apple drink (∼7.5 mmol nitrate) with apple juice on 24-h systolic or diastolic BP, and found no significant effect, although they reported a non-significant ∼4 mmHg difference between active and placebo group 6 h after consumption (Coles and Clifton, 2012). We found minimal effects of nitrate-containing beetroot juice on brachial systolic BP, so our data suggest that measurement of brachial BP as opposed to aortic BP is likely to have underestimated the effect of inorganic nitrate in some previous studies. Siervo et al. (2013) conducted a meta-analysis and systematic review of 12 studies using beetroot juice supplementation and demonstrated a change in brachial systolic BP of -4.5 mmHg (95% CI: -6.4, -2.5; *p* < 0.001) with nitrate doses ranging from 5.1 to 45 mmol. In the meta-regression these researchers carried out, the mean differences in systolic BP were not correlated with study duration. In keeping with our findings, ambulatory BP was not lowered by beetroot juice in the two studies of normotensive individuals included in the systematic review (Coles and Clifton, 2012; Hobbs et al., 2012).

Our study has strengths and limitations. A randomized double-blind placebo control study is a robust design; the sample size is small but adequate to detect a clinically important reduction in BP. Many previous studies have not been blinded as low-nitrate water or isomolar potassium and sodium chloride solutions have been used as the placebo (Larsen et al., 2006; Webb et al., 2008; Kapil et al., 2010; Larsen et al., 2010; Bahra et al., 2012; Hobbs et al., 2012). Our data are limited in that they were conducted in young healthy individuals receiving a single dose of beetroot juice and may not generalize to older people with or without high BP, or to other dosing regimens or durations of administration. Additionally, our study only considered the hypotensive effect of inorganic nitrate in beetroot juice. A recent meta-analysis (Bahadoran et al., 2017) has provided evidence that beetroot juice may contain factors other than nitrate that lower BP; given the design of our study it is not possible to conclude anything about the effect of other factors in beetroot juice on BP.

It has been suggested that beetroot juice may have value as a population-based intervention to lower BP, although our data showing an apparently short duration of BP lowering raise questions regarding its usefulness in younger normotensive individuals. Nevertheless, even small reductions in BP could lead to a large reduction in incident cardiovascular disease on a population level (Strachan and Rose, 1991). Dietary approaches to BP reduction could be useful in the face of the rising incidences of both obesity and diabetes. It remains to be established whether dietary supplementation with beetroot juice or other nitrate-rich vegetables, extracts or juices, is an effective, safe and acceptable method of population-scale BP reduction.

## Conclusion

In conclusion, nitrate-contained in beetroot juice lowered aortic systolic BP in normotensive individuals 30 min post-ingestion with minimal effects on brachial BP. This effect was of comparatively short duration and did not persist over 24-h. Previous studies only measuring brachial BP and not aortic BP may have underestimated the effects of inorganic nitrates on BP.

## Data Availability

The raw data supporting the conclusions of this manuscript will be made available by the authors, without undue reservation, to any qualified researcher.

## Author Contributions

AH and NC conceived and designed the study. SK and EC collected the data. SK, EC, and TT performed the analysis of the data. H-MD and TT undertook the statistical analysis. SK and AH wrote the paper with critical revisions from all authors. All authors provided approval for publication of the content and agree to be accountable for all aspects of the work in ensuring that questions related to the accuracy or integrity of any part of the work are appropriately investigated and resolved.

## Conflict of Interest Statement

The authors declare that the research was conducted in the absence of any commercial or financial relationships that could be construed as a potential conflict of interest.
