# Supplementary material for: A Double-Blind Placebo-Controlled Crossover Study of the Effect of Beetroot Juice Containing Dietary Nitrate on Aortic and Brachial Blood Pressure Over 24 h
Source: Front Physiol. 2019 Feb 4;10:47. doi: 10.3389/fphys.2019.00047 (PMC6369216; doi:10.3389/fphys.2019.00047)
Supplement: Supplementary file 1 [file Data_Sheet_1.docx]

**Supplementary Figures S1-S6**


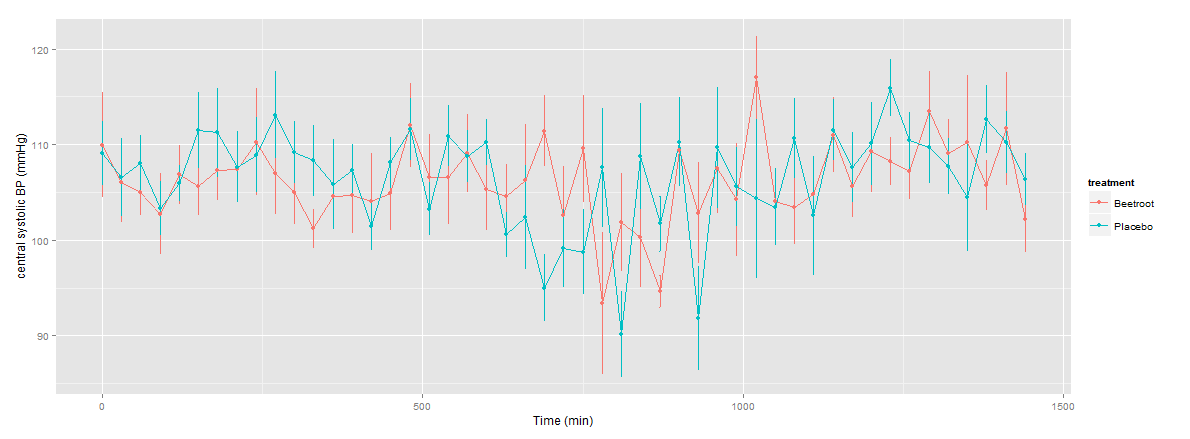


120

110

100

90

0

500

1000

1500

Time, mins

Aortic systolic blood pressure, mmHg

**Figure S1.** 24-hour ambulatory measures of aortic systolic blood pressure. Active beetroot (+nitrate) is shown in red, beetroot placebo in green. Data are means ± 95% confidence intervals.


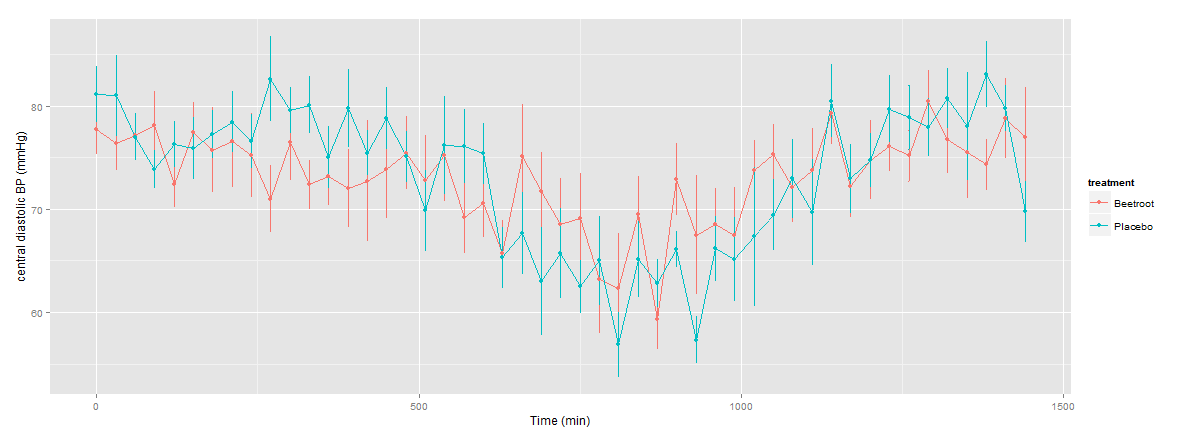


0

500

1000

1500

Time, mins

80

70

60

Diastolic blood pressure, mmHg

**Figure S2.** 24-hour ambulatory measures of diastolic blood pressure. Active beetroot (+nitrate) is shown in red, beetroot placebo in green. Data are means ± 95% confidence intervals.


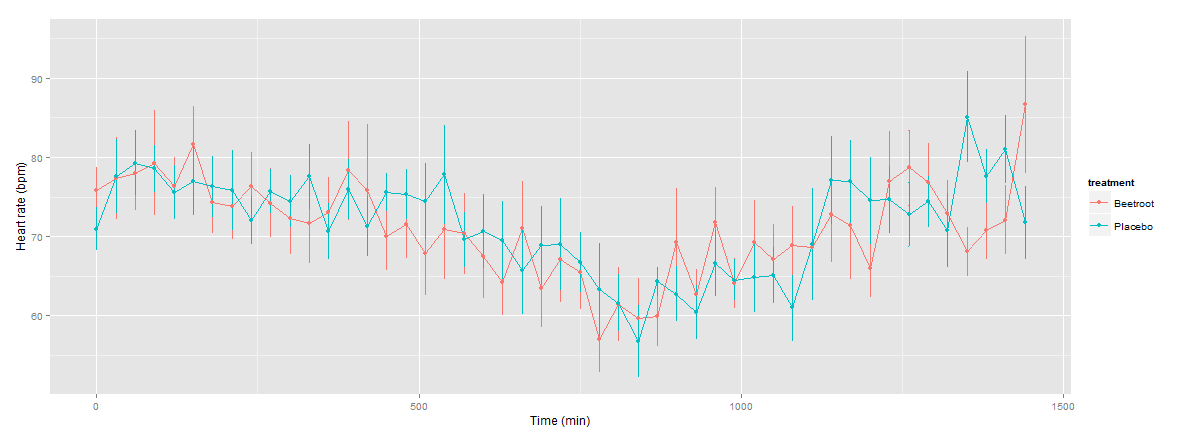


90

80

70

60

0

500

1000

1500

Time, mins

Heart rate, bpm

**Figure S3.** 24-hour ambulatory measures of heart rate. Active beetroot (+nitrate) is shown in red, beetroot placebo in green. Data are means ± 95% confidence intervals.


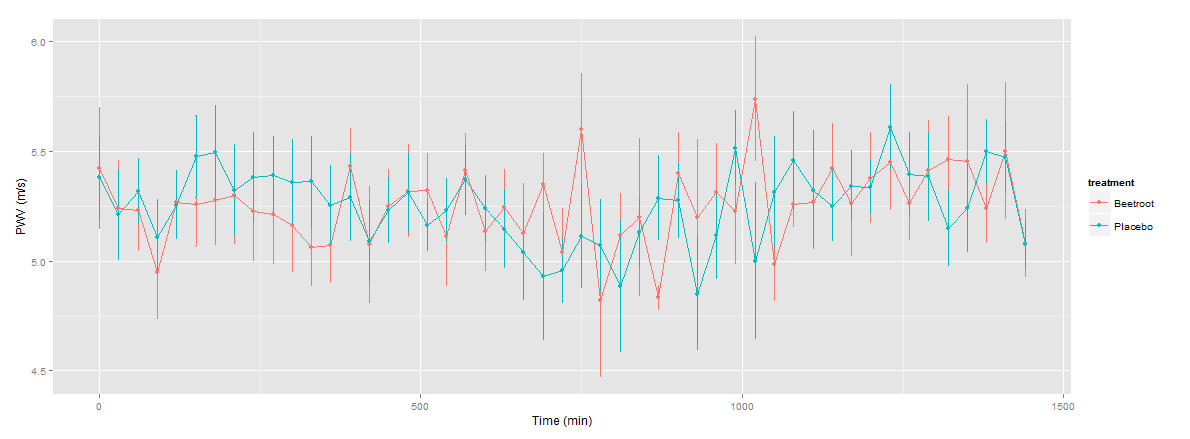


0

500

1000

1500

Time, mins

6.0

5.0

4.5

Pulse wave velocity, m/s

5.5

**Figure S4.** 24-hour ambulatory measures of pulse wave velocity (based on blood pressure cuff measurements). Active beetroot (+nitrate) is shown in red, beetroot placebo in green. Data are means ± 95% confidence intervals.


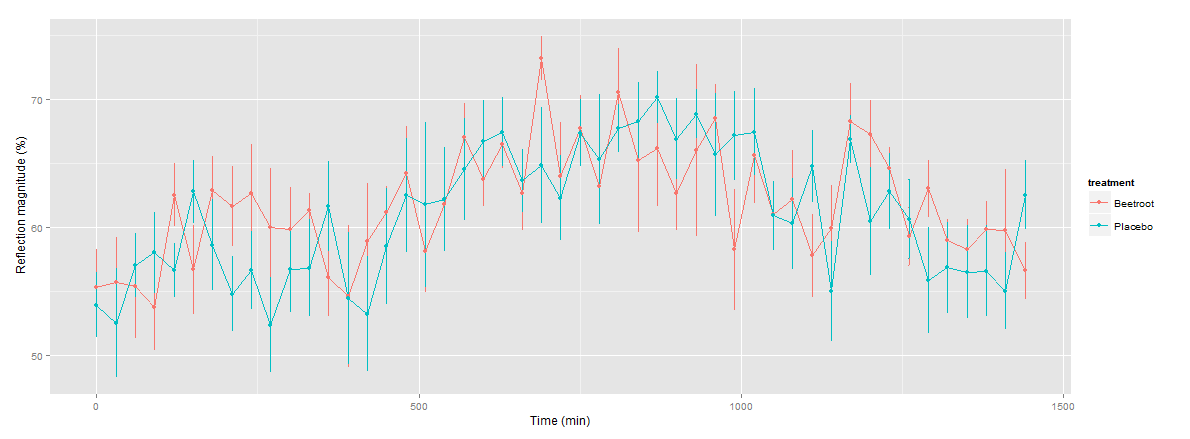


70

60

50

0

500

1000

1500

Time, mins

Reflection magnitude, %

**Figure S5.** 24-hour ambulatory measurement of reflection magnitude. Active beetroot (+nitrate) is shown in red, beetroot placebo in green. Data are means ± 95% confidence intervals.


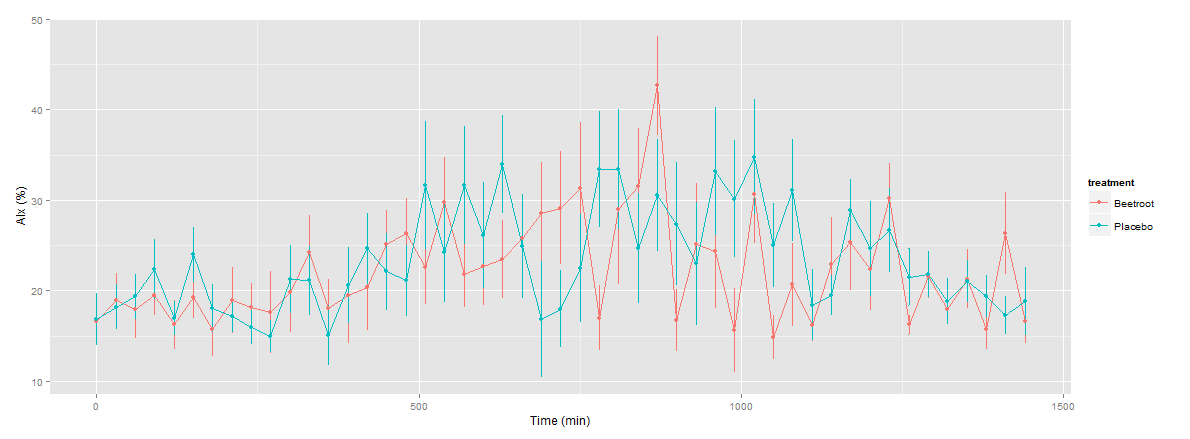


0

500

1000

1500

Time, mins

50

40

30

Augmentation index, %

20

10

**Figure S6.** 24-hour ambulatory measurement of augmentation index. Active beetroot (+nitrate) is shown in red, beetroot placebo in green. Data are means ± 95% confidence intervals.
